# The Study of Factors on the Small and Medium Enterprises' Adoption of Mobile Payment: Implications for the COVID-19 Era

**DOI:** 10.3389/fpubh.2021.646592

**Published:** 2021-03-16

**Authors:** Tianming Cao

**Affiliations:** Bidding and Material Procurement Center, Nanjing Institute of Technology, Nanjing, China

**Keywords:** COVID-19, mobile payment, small and medium enterprises, SMEs, adoption factors

## Abstract

The coronavirus disease 2019 (COVID-19) pandemic pushes people looking for shopping alternatives, seeking to avoid handling cash in favor of a safe and quick mobile payment. At this juncture, this paper examines the determinants of the adoption of mobile payment services among small and medium enterprises (SMEs) in China. The study proposes four-dimensional factors (business factors, technological competence, environment, and consumers' intentions) based on the literature review findings to understand the challenges of adopting mobile payment. A questionnaire is designed to solicit information from the participants. The findings reveal that business factors, technological competencies of SMEs in China, and the environment positively influence mobile payment adoption. Consumer intention has almost no influence on the adoption of mobile payment. Potential implications for the COVID-19 era are also discussed.

## Introduction

The use of mobile devices to provide financial services is widely accepted in advanced countries. It affects communication culture and greatly impacts most commercial and non-commercial systems, including financial activities (1). Many researchers have developed an interest in exploring mobile payment services since their remarkable effects and acceptance by consumers and giant telecommunication companies, financial institutions, and small and medium enterprises (SMEs) (2, 3). Most SMEs have embraced Apple Pay, Samsung Pay, PayPal, WeChat Pay, Alipay, and China MTN mobile transfer. At this stage, mobile banking and Facebook Libra cryptocurrency are also increasing competition (4). Overall, mobile payment is a growing business.

China has the leading mobile payment infrastructure. The mobile payment usage rate globally takes the first position for 3 years by 2020, about 805 million users, occupying 85.7% of the internet users (5). The rise in mobile payment in the domestic economy also has a spillover effect on developed countries. For instance, over 50% of the merchants surveyed in the United States and the United Kingdom state that Chinese customers' flow increased after they provided Alipay access (5). Particularly, during the COVID-19 pandemic, there is a growing share of mobile payments adoption since there is a significant reduction in the physical usage of cash, credit cards, and debit cards (6).

China's customers' usage rate is not much seen in SMEs (7). To enhance mobile payment understanding of SMEs, Shao et al. (8) indicate that a mobile payment requires a higher technological innovation than classical payment methods. Most developing countries lack technology. Therefore, despite the positive effects of mobile payment, many SMEs face challenges in applying the mobile payment system.

This study aims to examine key factors that hinder the adoption of mobile payment services among SMEs and compare and contrast the mobile payment initiative in China to a technologically advanced country, China. We also offer both practical and theoretical implications for SMEs for the coronavirus disease 2019 (COVID-19) pandemic. This study also offers several suggestions to SMEs in China, government institutions, and agencies. At this stage, policymakers and management of companies or institutions using mobile payment should make strategic decisions to combat the COVID-19 pandemic's future challenges. Given that China has the largest e-commerce and mobile payment, China's startup initiative can also consider the mobile payment demand's potential determinants.

The rest of the paper is organized as follows. *Literature Review and Theoretical Framework* provides a brief literature review and sets the theoretical framework. *Basic Features of Data and Findings* explains the survey data details, provides basic features of the collected data, and discusses the empirical findings. *Conclusion with Implications* concludes the paper by discussing potential implications.

## Literature Review and Theoretical Framework

### Previous Studies

A new paradigm has emerged under the mobile name payment (M-payment). Mallat (9) defines mobile payments as using a mobile device to conduct a payment transaction. According to Abrahão et al. (10), mobile payment systems provide flexibility, mobility, and efficiency to solve everyday problems or satisfy their users' wishes. Contribution to the M-payment definitions is transferring money to services or goods over mobile devices via Short Message Service (SMS), Browser, payment applications, and Quick Response (QR) code.

Mobile payment is also important for SMEs, but they can be defined according to different criteria in the various sectors: manufacturing, construction, and quarrying and mining sectors. For the retailing, miscellaneous, motor trades, and wholesale trades sectors, the sales turnover criterion is used. Whereas, the road transport sector uses several vehicles, the catering sector is based on ownership. In China, the definition of an SME is complex. It depends on a series of variables such as the industry it belongs to, its operating cost, its total assets, and its number of employees. SMEs constitute an overwhelming majority of the enterprises in China. They are the main part of economic development, as they represent 99.6% of China's companies, offer more than 80% of the job positions, and hold more than 70% of the patents (5).

Mobile payment system is becoming a leading payment method in developed economies (3) and developing markets such as China and Malaysia (11). Mobile payments further intensified the ease of online transactions (12). The introduction of innovative forms of Information and Communications Technology (ICT) services by SMEs to deliver financial services to all population segments is crucial for improving previous business processes. The ability to optimize ICT use has become a major criterion for promoting and enhancing future economic mobility in developing countries. However, the mobile payment system's tremendous benefits for SMEs have not been fully utilized in various emerging economies (13). Mun et al. (14) also found out that the perceived credibility (PC) is significantly associated with consumers' intention to use mobile payment services. The authors defined the PC as the consumers' judgment on mobile payment services' privacy and security issues. However, some authors also highlight that the usability problems are responsible for the low adoption of various payment systems (15, 16). Karsen et al. (17) opinioned that mobile devices should be used for payment by an authentication system to ensure every transaction's safety and comfort. Mobile payment service in China is a technological innovation product, which is the force of industrial evolution (18). Most of the developing countries are far from mobile payment development since they lack the required human capital, technological, environmental, and organizational requirements to adopt mobile payment for their SMEs (19). Dahbi and Benmoussa (20) reviewed the mobile payments adopting factors and categorized them into several headings such as organizational, technological, environmental, financial, and sociocultural basis. The authors conclude that security, trust, usability, government regulations, and institutional factors are the leading determinants of mobile payment adoption.

This paper aims to fill in the empirical literature gap by examining mobile payment determinants in China. Our novelty of the research is that we provided a survey in 2020, which captures the COVID-19 era. Therefore, our results may be interesting due to the changing pattern of the country's payment habits.

### Theoretical Framework and Hypothesis

The study proposes four-dimensional factors based on the literature review findings to understand the challenges of adopting mobile payment. The main factors are institutions, technology, consumers' intentions, and business unit or SMEs. These four variables define the critical path for analysis, and organizations need to consider them when adopting a mobile payment system.

The institution that could interplay in the adoption of mobile payment includes banks or financial corporations, internet service providers, telecommunication networks, trading partners, software applications, and government support. The extended unified theory of acceptance and use of technology (UTAUT2) as a basic model to accomplish the objectives (21). Besides, perceived usefulness, easy access, and mobile payment usage are considered under technology acceptance. SMEs proactively measure the outcome of adoption, cost and managerial implications, and the technical experts needed to accomplish mobile payment services.

Measures with proven reliability and validity are applied to operationalize the variables of the research model. The items are rated on a 5-point Likert scale, ranging from “strongly disagree” to “strongly agree” (see Table 1). Table 2 also explains the measurement variables, sources of the variables, and theoretical foundations.

**Table 1:** Measuring scale.

**Strongly Disagree (SD)**	**Disagree (D)**	**Neutral (N)**	**Agree (A)**	**Strongly Agree (SA)**
1	2	3	4	5

**Table 2:** Measurement variables, sources, and theoretical foundations.

**Factors**	**Items**	**Sources**	**Theoretical basis**
Business Factors	Customer Preferences Security, effort needed, management and employees level of knowledge, size of business, cost implications	Chhonkera et al. (18) and Kujala et al. (22)	Expectancy Theory
Technological Competence	Protection of privacy, instant transaction confirmation, familiarity of payment, system, software applications, internet connectivity and speed, security of transactions	Chhonkera et al. (18)	Unified Theory of Acceptance and Use of Technology (UTAUT)
Environment	Regulatory policies and support, incorporated payment facilities available, access to internet facilities and its price, language options available and literacy, institutional factors, government regulations, pressure from trading partners, pressure from competitors	Shao et al. (8)	Institutional Theory
Consumers Intention	Aware of mobile payments, the usefulness of mobile payments, use of electronic payments, transparency of transaction, authentication of payment, transaction confidentiality, optimistic, trust, usability etc.	Chhonkera et al. (18) and Kujala et al. (22)	Consumer Behavior Theory

Based on the above analysis, four hypotheses are given as the following:

H1: Business factors have a positive influence on the adoption intention.
H2: Technological competence has a positive influence on the adoption intention.
H3: Environment has a positive influence on the adoption intention.
H4: Consumer intention has a positive influence on the adoption intention.

## Basic Features of Data and Findings

### Population Data and Sample Size

Many review studies have considered the inclusion and exclusion criteria so far irrespective of research fields (18). The researcher purposively targeted five small and medium businesses in China for the first set of the questionnaire. Three main criteria were used: (1) must be in operations for more than 5 years, (2) must have licensed and legally recognized with no bad history, and (3) must be either B2B or B2C business type. This issue helps the researcher satisfy the research model and obtain all necessary information for further analysis. One hundred and eight respondents participated in the first section.

Users of mobile payments and businesses were invited to participate in the study. The businesses include delivery agencies, malls, KFC, McDonald's, etc. Generally, the study obtains information from 90 respondents in China and 90 respondents in other Chinese regions (Hong Kong, Macau, and Taiwan) for the second set.

Data are collected by conducting a field survey questionnaire from participants. The questionnaire has five sections: background information of respondents, institutional or external factors, technological factors, consumer behaviors, and business unit analysis. Two sets of questionnaires are prepared in English and Chinese. Before the questionnaire is administered, it is screened. A pilot test is conducted by sending the questionnaire to five friends to assess flexibility, grammatical errors, and appropriateness. Afterward, the questionnaire is reviewed and arranged to devoid of unethical issues.

The data are taken using the WeChat survey application. The questionnaires are filled in the app with due diligence. The following settings are done to ensure high reliability and validity of the data: (1) the app is set to anonymous, (2) respondents answers are strictly private, (3) response cannot be resubmitted once submitted, and (4) all questions are mandatory therefore a full set of data are obtained in every submission.

After designing the online questionnaire and cross-checking everything, it was posted in WeChat groups of business-oriented background. Clear instruction and confidentiality information accompanied the post. The first set of questions are administered to Chinese people only. This survey lasts for 2 weeks, and a total of 180 responses are received. After 3 days, the second part or set of questions is administered. The paper analyzes 180 responses for the second questionnaire analysis. Online data is obtained from the WeChat survey App.

### Background Information of the Respondents

According to the research questionnaire, the reliability and validity of the primary structure data of the respondents' background information are analyzed. The respondents' background information is gender, age range, educational qualifications, type of business engagement, and working years.

Table 3 illustrates the gender of the respondents; 69.4% are male, and 30.6% are female. Gender disparity does not influence the results of the study.

**Table 3:** Gender.

**Item**	**Frequency**	**Percent (%)**
Male	195	69.6
Female	85	30.4
Total	280	100.0

Table 4 shows the age range of the respondents. The majority of the participants are 21–30 years old, and they represent 69.4%. Those within 31–40 years were 30 representing 27.9%. Only five respondents were below 20 years, and none had more than 50 years of age.

**Table 4:** Age Range.

**Item**	**Frequency**	**Percent (%)**
Below 20	5	1.8
21–30	194	69.3
31–40	78	27.8
41–50	3	1.1
Total	280	100.0

Educational level of staff and owners of small business contributes to M-payment adoption. Table 5 shows that 75 of the respondents, constituting 69.4%, were in their postgraduate level, while 29.6% had college or university education. High school was 0.9%, and none had no formal education.

**Table 5:** Educational background of respondents.

**Items**	**Frequency**	**Percent (%)**
High school	3	1.1
College/university	83	29.6
Postgraduate	194	69.3
Total	280	100.0

Table 5 shows the educational background of respondents; postgraduate accounts for 69.3%, which shows that the respondents are highly educated,

The respondents' sector of business engagement is shown in Table 6. Most of the respondents are students, accounting for 69.4%, doing business is the smallest part, only accounting for 2.8%.

**Table 6:** Sector of businesses.

**Item**	**Frequency**	**Percent (%)**
Private/company	21	7.4
Public	18	6.5
Self-employed	39	13.9
Student	194	69.4
Yet to do business	8	2.8
Total	280	100.0

Table 7 indicates the years of work. One hundred and sixteen of the respondents had between 11 and 15 years, representing 41.4%. This evidence is followed by 6–10 years, 65 respondents, and it represents 23.2%.

**Table 7:** Years of working.

**Item**	**Frequency**	**Percent (%)**
Below 5	60	21.4
6–10	65	23.2
11–15	116	41.4
16–20	34	12.2
21 above	5	1.8
Total	280	100.0

### Reliability and Validity Analysis

The main construct variables affecting mobile payment adoption by SMEs are business factors, technological competence, environment, and consumer intention. The reliability and validity of the scale are analyzed using the valid questionnaire data collected. The reliability and validity of the information of each measurement scale are calculated as shown in Table 8.

**Table 8:** Reliability and validity.

**Variable**	**Cronbach α**	**KMO**	**Bartlett's sphericity test**			**Cumulative total variance**
			**Bangla**	**df**	**Sig**.	
Business Factors (BF)	0.768	0.726	227.846	6	0.000	52.682%
Technological Competence (TC)	0.634	0.688	121.545	3	0.000	55.859%
Environment (EN)	0.698	0.646	116.343	3	0.000	54.841%
Consumer Intention (CI)	0.683	0.722	258.476	3	0.000	56.443%
Adoption Intention (AI)	0.742	0.716	352.358	6	0.000	55.887%

It can be seen from Table 8 that the Cronbach α of each scale is >0.63, and the total variance of the cumulative interpretation is above 52%, indicating that the questionnaire has certain credibility in the study. The results of the confirmatory factor analysis using Mplus were chi-square = 192.895, comparative fit index (CFI) = 0.938, Tucker–Lewis index (TLI) = 0.929, and root mean square error of approximation (RMSEA) = 0.082. This model fits well according to the current criteria, showing that the research measurements are four independent variables.

### Descriptive Statistical Analysis

The average value, standard deviation, and correlation coefficient between each measured variable are calculated as shown in Table 9.

**Table 9:** Descriptive statistics and correlation coefficients.

**Variable**	**M**	**SD**	**Business factors**	**Technological competence**	**Environment**	**Consumer intentions**
Business factors	3.294	0.829				
Technological competence	3.628	0.848	0.596***			
Environment	3.625	0.821	0.474***	0.586***		
Consumer intentions	3.454	0.835	0.675***	0.683***	0.583***	
Adoption intention	3.545	0.832	0.688***	0.636***	0.576***	0.642***

Significance level ***means 0.1%.

Business factors, technological competence, environment, and consumer intentions are significantly and positively correlated, indicating that they are all factors affecting adoption. Business factors is significantly positively correlated with the technological competence, environment and customer intention. The above provides the necessary preconditions for analyzing the relationship between the variable.

Then, the data validity test of Kaiser–Meyer–Olkin (KMO) and Bartlett is performed based on SPSS. KMO test is a measure of how suited the data are for analysis. The test measures the sampling adequacy for the variables in the model. KMO sampling adequacy is 0.824, which is meritorious. Bartlett's test of sphericity is a <0.05 (Sig. 000). The chi-square results are significant χ^2^ = 880.889. This test and the Cronbach alpha reliability test validate the data for all further analysis (see Table 10).

**Table 10:** KMO and Bartlett's test.

**Test**		**Result**
Kaiser–Meyer–Olkin Measure of Sampling Adequacy		0.824
Bartlett's Test of Sphericity	Approx. chi-square	880.889
	df	210
	Sig.	0.000

### Factors That Hinder Mobile Payment Adoption Among SMEs in China

This article divides the factors that influence mobile payment adoption challenges among Chinese SMEs into four main categories to investigate how each field and some variables affect SMEs carefully. This analysis covers factors that influence SMEs in adopting a mobile payment service system. It includes cost of adoption, employees' education level, business types, mobile payment readiness, and technology competency (21, 23).

#### Business Factors

The business or SMEs' characteristics that effectively hinder the business idea of accepting mobile payment service in China are revealed and illustrated in Table 11.

**Table 11:** Descriptive statistics of business factors.

**Variable Description**	***N***	**Mean**	**Std. Deviation**	**Variance**
Cost of adoption	280	3.90	1.046	1.145
Education level of employees	280	3.83	0.786	0.621
Type of business	280	3.40	1.251	1.586
Business readiness	280	4.15	0.634	0.424

Table 11 shows the mean, standard deviation, and average variance of business or SMEs factors that hinder mobile payment adoption in China. Regarding the cost of adoption, the respondents agree or strongly agree that it significantly militates against mobile payment services. The average cost of adoption is 3.90. Implicitly, all the business factor category figures were wholly accepted and reaffirmed that it indeed hinders SMEs' proposal to practice mobile payment system. The highest item is business readiness of SMEs (4.15); education level of employees is 3.83, and the lowest average level is business 3.40.

#### Technological Competence

SMEs that have implemented mobile payment system and others that have the idea to practice cannot swerve from technological issues. A survey is conducted to analyze which technological issues affect the Chinese SMEs' attempt to adopt mobile payment (see Table 12).

**Table 12:** Descriptive statistics of technological factors.

**Variables**	***N***	**Mean**	**Std. Deviation**	**Variance**
Availability of internet connectivity and speed	280	4.09	1.054	1.083
Network connection among payment partners	280	4.33	0.861	0.756
Reliable software applications	280	3.98	1.066	1.118
Technological level of payment facilities	280	3.91	0.930	0.826
Compatibility of payment facilities available	280	4.32	0.902	0.818

Table 12 emphasizes that the technological factors are the key in impeding SMEs in mobile payment adoption. The most strongly agreed item is network connection among payment partners (4.33, strongly agreed). Network connection is a great challenge among SMEs since its good network connection is limited to a few urban areas, making it difficult for more business units to embrace mobile payment. The connection to bridge quality payment service among all the mobile payment partners suffer handicap. This item is followed by the availability of internet connectivity (4.09), where there is poor network; slow and unavailability of internet also exist. Moreover, the remaining factors are the reliable software applications (3.98), technological level of payment facilities (3.91), and compatibility of payment facilities available (3.86).

#### Environment

The external factors are challenges of other technology and own business limitations that hinder mobile payment adoption. These factors are sometimes beyond the SMEs' control and need government or industrial interventions to facilitate implementations.

Table 13 shows that the ICT infrastructure of China affects SMEs to adopt mobile payment services. This evidence was strongly agreed with a total value of (4.06). Another external factor is cooperation from telecom providers and banks (3.96). Most banks cannot provide security and trust effective transaction coupled with network connectivity challenges for mobile payment to be perfect.

**Table 13:** Descriptive statistics of environment.

**Description**	**Mean**	**Std. Deviation**	**Variance**
Regulatory policies and support by government	3.75	0.926	0.842
Cooperation from telecom providers and banks	3.96	0.918	0.825
Pressure from competitors and stakeholders	3.66	1.026	1.044
ICT infrastructure level in China	4.06	0.916	0.832
Perceived public awareness and compatibility	3.39	1.185	1.380
Language options available and literacy	3.88	1.036	1.053

#### Consumer Intention

The author investigated many consumer intentions factors, and the respondents reacted in different views. The result is tabulated in Table 14.

**Table 14:** Descriptive statistics of consumer factors.

**Description**	***N***	**Mean**	**Std. Deviation**	**Variance**
Familiarity and complexity to users	280	3.91	0.918	0.845
Perceived trust and security	280	4.25	0.856	0.724
Consumers readiness	280	3.85	0.848	0.716
Perceived ease and usefulness	280	3.96	0.796	0.628
Relative advantage	280	4.06	0.877	0.758

Table 14 depicts the consumer aspects that limit SMEs for adopting mobile payment system. The most strongly agreed factor from the respondents is “perceived trust and security” (4.25). With mobile, internet, and bank fraud in China community, consumers find it difficult to believe in the mobile payment system. However, the m-payment system could be successful depending on the relative advantage over cash payment (4.06). “Perceived ease and usefulness” (3.96) and “familiarity and complexity to users” (3.91).

### Hypothesis Verification

**Figure 1:**
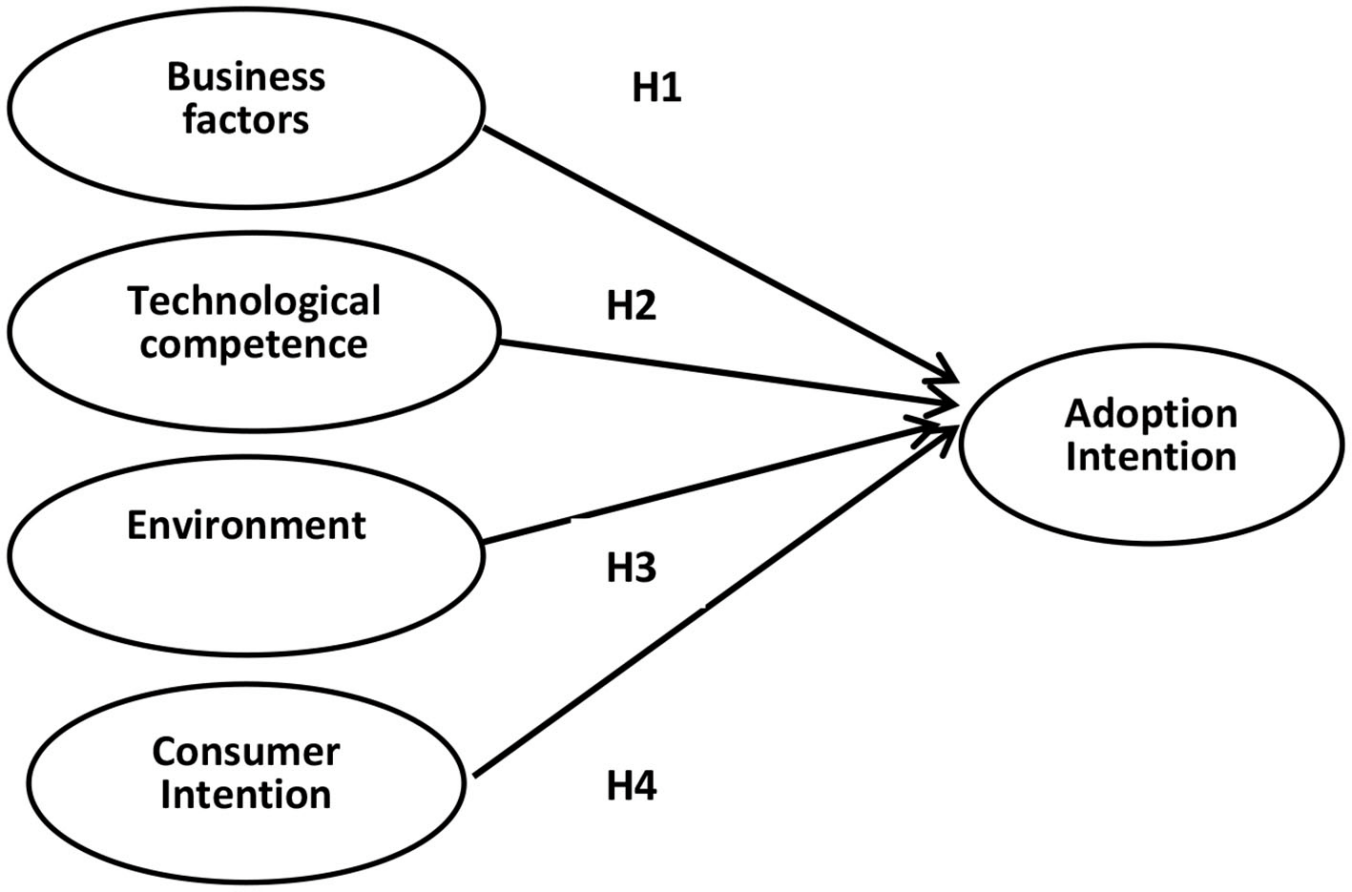
Proposed four-dimensional model.

**Figure 2:**
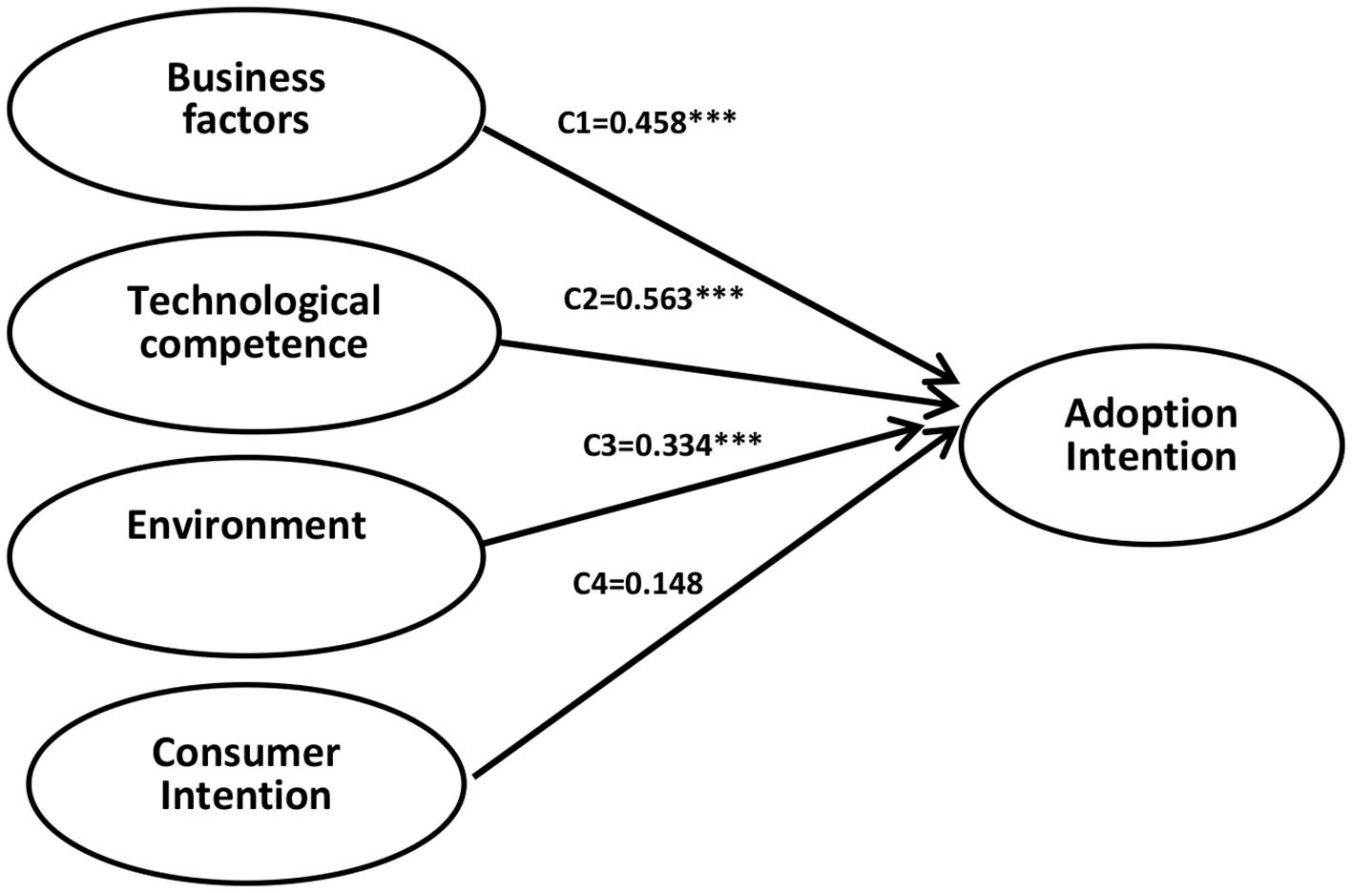
Test of hypothesis. Significance level ***means 0.1%.

This paper uses Mplus7.0 to perform path analysis and hypothesis testing. According to the above test procedure, a relationship model is established between business factors, technological competence, environment, consumer intention and adoption intention (see Figure 1). The test results are shown in Figure 2.

Since chi-square = 0, df = 0, CFI = 1.000, TLI = 1.000, RMSEA = 0.000, standardized root mean square residual (SRMR) = 0.000, the model fits well. As can be seen from Figure 2, business factors significantly predict the adoption intention (c1 = 0.458, t = 10.932, *p* = 0.000), assuming H1 is verified. Technological competence significantly influences adoption intention (c2 = −0.563, t = −8.611, *p* = 0.000), indicating that the technological competence predicts adoption intention, assuming H2 is verified. Environment also influences adoption intention (c2 = −0.334, t = 12.082, *p* = 0.000), indicating that environment is also a key factor influencing the adoption intention, then H3 is verified. Besides, customer intention may have no influence on adoption intention (c4 = −0.148, t = −1.018, *p* = 0.067), then H4 is not verified. Business factors, technological competence, and environment positively influence mobile payment's adoption intention, but the SMEs' customer intention does not influence the adoption intention (22).

## Conclusion With Implications

The COVID-19 pandemic is expected to have a significant effect on the payment card market. Contactless payment is tagged as a more hygienic and safer way of making payments (24). This issue promotes mobile payment. The COVID-19 pushes people to look for alternative payments in shopping and seeking to avoid handling cash and other materials to safely and quickly check out. However, as the COVID-19 enhances mobile payments, it is important to secure online and digital transactions (4). At this stage, the SMEs problems have posed many challenges for mobile payment service adoption in China. This study reveals that “business factors, technology competence of SMEs in China, mobile payment environment form major factors that influence mobile payment acceptance.”

The questionnaire results reveal important observations and emphasize that technological factors impede SMEs in mobile payment adoption. The most strongly agreed item is “network connection among payment partners.” Another challenge is the poor and slow internet connectivity; moreover, reliable software applications, technological level of payment facilities, and compatibility of payment facilities are available.

Some of the main external factors that affect the adoption of mobile payment systems by China small- and medium-sized enterprises revealed by this study are ICT infrastructure in China and cooperation from telecom providers and banks. Most banks cannot secure and trust effective transactions and network connectivity challenges for mobile payment to be perfect. The external environment factors do not facilitate mobile payment and are not ready due to the large technological and network gap. The basic infrastructure to incorporate all the mobile payment partners seems too expensive, thereby discouraging China's payment system.

We investigated many consumer intentions factors, and the respondents reacted in different views. The most strongly agreed factor from the respondents is “perceived trust and security.” With mobile, internet, and bank fraud in Chinese community, consumers find it difficult to believe in the mobile payment system. However, the m-payment system could be successful depending on the relative advantage over cash payment. According to the technology acceptance model (TAM) model, it suggests that perceived usefulness and ease of use are the determinants of users' willingness to accept and use systems. This evidence implies that consumers' perceived usefulness of mobile payment over cash shows a positive emotional enjoyment and will be accepted if SMEs implement mobile payment adoption.

Based on the study's findings, we recommend the following for SMEs management, owners, government, and other mobile payment partners. In technology, internet, and network connections, the national communication authority should quickly find reliable measures to offer quality internet access and good network coverage connection in collaboration with the private network providers. China should also embark on ICT infrastructure to facilitate a mobile payment system. Management of SMEs or business units should train their staff to acquire more relevant mobile payment system knowledge. In addition, we suggest that SMEs seek credit to facilitate the cost of mobile payment adoption and upgrade their technological equipment on business factors. Future studies can focus on the post-COVID-19 era for analyzing the determinants of mobile payment adoption in other developing countries.

## Data Availability Statement

The raw data supporting the conclusions of this article will be made available by the authors, without undue reservation.

## Ethics Statement

The studies involving human participants were reviewed and approved by The survey in the study has been approved by the ethics committee of the Nanjing Institute of Technology. The patients/participants provided their written informed consent to participate in this study.

## Author Contributions

The author confirms being the sole contributor of this work and has approved it for publication.

## Conflict of Interest

The author declares that the research was conducted in the absence of any commercial or financial relationships that could be construed as a potential conflict of interest.
